# A label-free impedimetric strategy using interdigitated electrodes for tetracycline detection in Milk

**DOI:** 10.1016/j.fochx.2026.104171

**Published:** 2026-07-06

**Authors:** Son Hai Nguyen, Huy Quang Nguyen, Mai Thi Tran

**Affiliations:** a School of Mechanical Engineering, Hanoi University of Science and Technology, Hanoi, Viet Nam; b College of Engineering and Computer Science, VinUniversity, Hanoi, Viet Nam

**Keywords:** Interdigitated electrodes, Mn-doped ZnS, Chitosan, Impedimetric biosensor, Tetracycline detection, Milk safety

## Abstract

The extensive use of tetracycline in livestock production has resulted in residues in dairy products, necessitating analytical methods that are accurate, robust, and compatible with complex food matrices. This work presents a label-free, non-enzymatic impedimetric sensor based on interdigitated electrodes (IDEs) for quantitative tetracycline detection in milk. Electrochemical impedance spectroscopy is used to monitor interfacial charge-transfer processes, emphasizing practical analytical translation rather than the development of new nanomaterials. The IDE-based sensor exhibits a signal-on response, with charge-transfer resistance decreasing monotonically as tetracycline concentration increases. Using Nernst-type linearization, the sensor achieves a linear range of 62.5–1000 nM, with limits of detection and quantification of 2.47 nM and 8.23 nM, respectively. High accuracy in spiked milk samples (relative error < 5%), strong matrix tolerance, good analytical discrimination, reproducibility, and stability demonstrate the sensor's practical potential for routine tetracycline residue screening in dairy products.

## Highlights

• Interdigitated electrode-based impedimetric strategy enables sensitive tetracycline detection in milk
• Label-free and non-enzymatic quantification of tetracycline residues in complex dairy matrices
• Wide linear response (62.5–1000 nM) with low detection (2.47 nM) and quantification (8.23 nM) limits
• High selectivity against common coexisting antibiotics and interferents in milk
• Reproducible and stable sensing platform suitable for practical food analysis applications

## 1 Introduction

The widespread use of tetracycline (TET) antibiotics in livestock production, particularly in dairy farming, has raised persistent concerns about food safety, antimicrobial resistance, and broader public-health risks (Alabi et al., 2025). Experimental studies have also reported potential dose- and time-dependent reproductive effects of TET exposure, including disruption of the testicular microenvironment and reduced testosterone levels (Popoola et al., 2014). These concerns have become more pronounced in recent years, as increased pharmaceutical consumption during the COVID-19 pandemic has contributed to antibiotic residues in aquatic environments, with potential implications for the food chain (Sosa-Hernández et al., 2021). Addressing pharmaceutical contamination requires complementary strategies for both rapid detection and effective removal. In this context, nanomaterials have also been widely investigated as photocatalysts for the degradation of persistent pharmaceutical residues in water (Ebrahimi & Akhavan, 2022).

Within the food-chain context, milk is a particularly important matrix because residual TET may disrupt the gut microbiome, provoke allergic responses, and accelerate the emergence of antibiotic-resistant pathogens, posing risks to both consumers and healthcare systems (Shayista et al., 2025). To mitigate these risks, international regulatory bodies such as FAO/WHO have established strict maximum residue limits (MRLs) for tetracyclines in milk (100 μg/L) (FAO, 1998). However, effective routine monitoring at dairy farms and processing facilities remains challenging due to the limited availability of rapid, cost-effective, and field-deployable analytical tools.

Conventional chromatographic techniques, including high-performance liquid chromatography (HPLC) and LC-MS/MS, offer high sensitivity and selectivity for the analysis of antibiotic residues. Nevertheless, their reliance on sophisticated instrumentation, extensive sample preparation, and long analysis times restricts their suitability for real-time or on-site monitoring applications (Hu et al., 2024; Peris-Vicente et al., 2022). In this context, electrochemical biosensors have emerged as attractive alternatives due to their low cost, short response times, portability, and compatibility with miniaturized electronics. Among these platforms, electrochemical impedance spectroscopy (EIS) is particularly appealing because it enables label-free interrogation of interfacial charge-transfer processes, offering high sensitivity to subtle physicochemical changes induced by analyte interactions (Nguyen et al., 2023).

The analytical performance of electrochemical biosensors is strongly influenced by the structure and surface chemistry of the electrode-solution interface. Recent studies have demonstrated that nanostructured sensing interfaces can substantially improve electrochemical transduction by enhancing interfacial charge-transfer kinetics, increasing accessible electroactive sites, and amplifying surface-confined signal responses (Akhavan et al., 2012; Heidarimoghadam et al., 2016; Nguyen et al., 2024b; Štukovnik et al., 2023). Zinc sulfide (ZnS) nanomaterials have attracted growing interest in this regard owing to their tunable optoelectronic properties, chemical stability, and compatibility with polymeric matrices (Nguyen et al., 2024a; Nguyen & Tran, 2024). Furthermore, incorporation of transition-metal dopants such as Mn^2+^ into ZnS can introduce defect states, enhance electrical conductivity, and increase surface reactivity, thereby improving electrochemical signal transduction (Othman et al., 2021). Chitosan (CH) is a naturally derived biopolymer containing abundant amino and hydroxyl groups and is well known for its biocompatibility, biodegradability, hydrophilicity, tunable crosslinking chemistry, and film-forming ability (Jiménez-Gómez & Cecilia, 2020; Saeedi et al., 2022). These properties make chitosan a suitable functional matrix for dispersing and immobilizing nanoparticles on electrode surfaces and may facilitate analyte-surface interactions through hydrogen bonding and possible coordination interactions.

To effectively translate these interfacial advantages into practical analytical devices, interdigitated electrodes have emerged as a powerful transducer architecture for impedimetric sensing. The microscale interelectrode spacing of IDEs generates confined electric fields that enhance sensitivity to small perturbations at the solid-liquid interface, enabling accurate detection of low-abundance analytes (Kumar et al., 2024). In addition, IDEs support miniaturization, low sample consumption, rapid response, and cost-efficient mass fabrication using established microfabrication techniques. Recent reports have demonstrated that IDE-based EIS sensors functionalized with nanostructured interfaces can achieve reliable, label-free quantification of antibiotic residues, including kanamycin, azithromycin, clarithromycin, and erythromycin, in food and environmental matrices (Magro et al., 2022; Zheng et al., 2025). These attributes make IDE-EIS platforms particularly well-suited for portable and on-site monitoring applications in food safety control.

Despite these advances, the analytical implementation of Mn-doped ZnS nanoparticles embedded within a chitosan matrix (Mn:ZnS-CH) on interdigitated electrodes for impedimetric detection of tetracycline in milk has not yet been systematically explored. In particular, the combined effects of Mn-induced defect states and chitosan-mediated interfacial adsorption offer a promising strategy to enhance charge-transfer modulation while maintaining compatibility with complex dairy matrices. This study therefore develops and evaluates a label-free impedimetric sensing platform based on Mn:ZnS-CH-modified interdigitated electrodes for rapid and quantitative tetracycline detection in milk. The study investigates the effect of the Mn:ZnS-CH interface on charge-transfer resistance, determines the sensor's working range and detection capability, evaluates its ability to discriminate tetracycline from selected potentially interfering antibiotics, and assesses its analytical accuracy in tetracycline-spiked diluted milk samples. By focusing on analytical validation in a real food matrix rather than on the development of a new sensing material, this work aims to establish a robust, scalable electrochemical strategy for tetracycline residue screening in dairy products.

## 2 Materials and methods

### 2.1 Chemicals and reagents

Tetracycline hydrochloride, amoxicillin (AMX), ampicillin (AMP), penicillin G (PCN), doxycycline (DOX), and cephalexin (CEX) were purchased from Shanghai Macklin Biochemical Co., Ltd. All chemicals were of analytical grade and used as received without further purification. Commercial organic cow milk was procured from a local market and stored at 4 °C prior to use.

### 2.2 Fabrication of Mn:ZnS-CH-modified IDE electrodes

ZnS nanoparticles doped with Mn and the corresponding Mn:ZnS-chitosan (Mn:ZnS-CH) nanocomposite were synthesized and comprehensively characterized in our previous studies (Nguyen and Tran, 2025a, Nguyen and Tran, 2025b). In the present work, these established materials were employed to construct an IDE-based impedimetric sensing interface, with the focus placed on analytical implementation rather than material development.

**Fig. 1:**
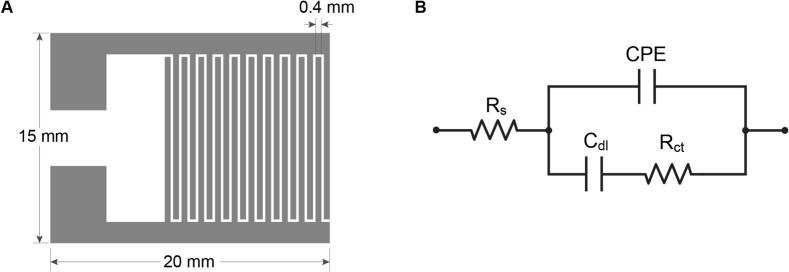
(A) Schematic illustration and geometric parameters of the interdigitated electrodes (IDEs). (B) The Randles circuit was used to fit all experimental data in this work.

The interdigitated electrodes exhibit a comb-like geometry consisting of 20 fingers, with a gap spacing of 200 μm and a finger width of 400 μm, as illustrated in Fig. 1A. The IDE substrates were fabricated from aluminum and subsequently coated with a thin gold layer to ensure stable electrochemical performance (T. N. P. Nguyen et al., 2024). Prior to surface modification, the gold IDEs were sequentially rinsed with ethanol, acetone, and deionized water, followed by drying under ambient conditions.

A defined volume of 10 μL of the Mn:ZnS-CH suspension was drop-cast onto the active area of each IDE and allowed to dry at room temperature. After coating, the electrodes were gently rinsed with deionized water to remove loosely bound material and then dried before electrochemical measurements, ensuring reproducible surface coverage.

All electrochemical impedance measurements were performed using a Hioki IM3536 impedance analyzer (Japan). EIS spectra were recorded over a frequency range of 4 Hz to 10 kHz using a sinusoidal AC perturbation of 10 mV at open-circuit potential. The resulting Nyquist plots were fitted using a Randles equivalent circuit (Fig. 1B), comprising a solution resistance (R_s_), a constant phase element (CPE), a double-layer capacitance (C_dl_), and a charge-transfer resistance (R_ct_). Among these parameters, R_ct_ was selected as the primary analytical signal for tetracycline detection.

### 2.3 Sample preparation and tetracycline detection procedure

Commercial organic milk was diluted with deionized water at a 1:30 ratio prior to analysis. This dilution ratio was selected to reduce matrix-induced interference, including viscosity effects, protein/fat adsorption, and nonspecific fouling at the IDE surface, while maintaining compatibility with the proposed sensor's working range. Tetracycline was subsequently spiked into the diluted milk samples to achieve final concentrations of 62.5, 125, 250, 500, and 1000 nM, corresponding to the working range of the proposed sensing platform. For each measurement, a fixed volume of the spiked sample was deposited onto the Mn:ZnS-CH-modified IDE surface and incubated for 5 min before electrochemical impedance spectroscopy (EIS) was performed.

The charge-transfer resistance R_ct_ extracted from the fitted impedance spectra was used as the primary analytical signal and plotted as a function of tetracycline concentration to construct calibration curves. Selectivity was evaluated by exposing the sensor to AMX, AMP, PCN, DOX, CEX, and glucose at concentrations identical to those used for tetracycline, and by comparing the corresponding R_ct_ responses to assess potential interference. Reproducibility was assessed using three independently fabricated electrodes prepared on different days under identical experimental conditions.

## 3 Results and discussion

### 3.1 Impedimetric detection of tetracycline in Milk using Mn:ZnS-CH electrodes

**Fig. 2:**
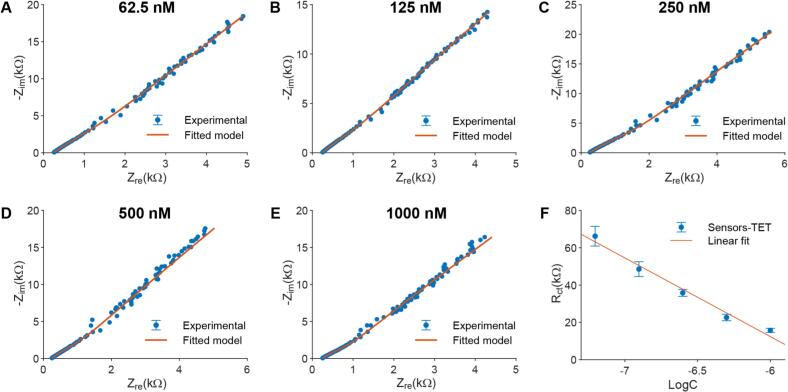
Nyquist plots of Mn:ZnS-CH–based biosensors in deionized water at different tetracycline concentrations: (A) 62.5 nM, (B) 125 nM, (C) 250 nM, (D) 500 nM, and (E) 1000 nM. The blue symbols represent the experimental data, and the red curves are the fitted responses obtained using the Randles equivalent circuit model shown in Fig. 1. (F) Charge transfer resistance R_ct_ of the biosensor as a function of the logarithm of tetracycline concentration, showing a linear calibration relationship. (For interpretation of the references to colour in this figure legend, the reader is referred to the web version of this article.)

The Mn:ZnS-chitosan-modified interdigitated electrodes (IDEs) exhibited a highly consistent and concentration-dependent impedimetric response upon exposure to tetracycline-spiked milk samples. As shown in Fig. 2, the Nyquist plots display well-defined semicircular features whose diameters decrease progressively with increasing tetracycline concentration, indicating a systematic reduction in the charge-transfer resistance. This behavior reflects a gradual acceleration of interfacial electron-transfer kinetics rather than random fluctuations associated with matrix effects, confirming that the sensing response is governed by analyte-induced interfacial modulation.

The observed monotonic decrease in R_ct_ provides a clear signal-on impedimetric mechanism, in which the presence of tetracycline enhances rather than suppresses charge-transfer processes at the electrode-electrolyte interface. This response contrasts with the signal-off behavior commonly reported for blocking-type biosensors, where analyte adsorption typically increases interfacial resistance by hindering electron transport. The signal enhancement observed here suggests that tetracycline molecules actively facilitate charge transport at the Mn:ZnS-CH interface, likely through adsorption-assisted electronic coupling and defect-mediated conduction pathways.

The fitted electrochemical parameters extracted from the Randles equivalent circuit are summarized in Table 1. Notably, while the solution resistance R_s_, constant phase element CPE, and double-layer capacitance C_dl_ remain relatively stable across the tested concentration range, R_ct_ exhibits a pronounced and systematic decrease, confirming that charge-transfer resistance is the dominant sensing parameter in this system. This selective sensitivity of R_ct_ further supports its suitability as a robust analytical signal for quantitative tetracycline detection in milk.

**Table 1:** Fitted parameters of proposed biosensors with TET.

C (nM)	R_s_ (Ω)	CPE (F)	n	C_dl_ (F)	R_ct_ (Ω)
1000	227.31 ± 4.52	1.62 × 10^−6^ ± 4.18 × 10^−8^	0.84 ± 0.004	1.10 × 10^−7^ ± 5.15 × 10^−9^	13,920.56 ± 1118.48
500	221.38 ± 5.25	1.61 × 10^−6^ ± 2.96 × 10^−8^	0.83 ± 0.003	8.74 × 10^−8^ ± 3.96 × 10^−9^	20,776.89 ± 1755.78
250	233.18 ± 3.13	1.32 × 10^−6^ ± 2.16 × 10^−8^	0.83 ± 0.003	8.26 × 10^−8^ ± 3.08 × 10^−9^	33,655 ± 1878.81
125	231.78 ± 3.41	2.11 × 10^−6^ ± 1.02 × 10^−7^	0.82 ± 0.010	6.77 × 10^−8^ ± 5.39 × 10^−9^	41,124 ± 3958.77
62.5	243.93 ± 3.24	1.47 × 10^−6^ ± 3.35 × 10^−8^	0.85 ± 0.002	5.64 × 10^−8^ ± 2.82 × 10^−9^	65,771 ± 5294.79

Quantitatively, the R_ct_ values decreased markedly from 66,209 ± 5295 Ω at 62.5 nM to 15,704 ± 1118 Ω at 1000 nM TET, corresponding to a 4.2-fold increase in the charge-transfer rate across the tested range. When R_ct_ was plotted as a function of the logarithm of tetracycline concentration, a linear calibration relationship was obtained over the range of 62.5–1000 nM, as described by Eq. (1):

(1) $$R_{\mathrm{ct}}=-4.22\times 10^{4}\text{logC}-2.41\times 10^{5}$$

with a high correlation coefficient of R^2^ = 0.98. The high linearity confirms that the Mn:ZnS-CH sensing interface reliably converts variations in tetracycline concentration into predictable changes in interfacial resistance, enabling quantitative analysis even in the chemically complex milk matrix.

To determine the limit of detection (LOD) and limit of quantification (LOQ), extrapolation artifacts associated with logarithmic calibration were avoided by following the approach reported in (Alberti et al., 2022). Specifically, a Nernst-type linearization was employed by plotting y = 10^Rct/slope^ as a function of concentration (C), which preserves proportionality between the analytical signal and concentration in the low-concentration regime and minimizes bias introduced by back-extrapolation of logarithmic calibration curves. The resulting linear regression yielded the following calibration equation (R^2^ = 0.959):

(2) y=0.359×C+0.0362

The LOD and LOQ were subsequently calculated using the 3σ/S and 10σ/S criteria, respectively, where σ denotes the standard deviation of the response and S is the slope of the calibration curve in Eq. (2). The resulting LOD and LOQ values of 2.47 nM and 8.23 nM demonstrate reliable nanomolar-level sensitivity in a real milk matrix, where protein adsorption, lipid content, and ionic background often obscure low-level electrochemical signals. These values correspond to approximately 1.10 and 3.66 μg/L, respectively, which are well below the regulatory MRL for tetracycline in milk of 100 μg/L, equivalent to approximately 225 nM. The LOD and LOQ should be regarded as analytical sensitivity parameters rather than screening decision thresholds. The high sensitivity of the sensor provides an analytical margin below the regulatory limit but does not, by itself, lead to false-positive classification. For routine screening, an appropriate cutoff should be established through matrix-matched method validation, taking into account analytical selectivity, matrix effects, measurement variability, and uncertainty. Samples yielding results at or above the validated cutoff should undergo confirmatory analysis using an established reference method.

### 3.2 Analytical discrimination of tetracycline

The analytical discrimination capability of the Mn:ZnS-CH-modified IDEs was systematically evaluated by comparing their impedimetric responses toward tetracycline with those toward structurally and functionally distinct antibiotics commonly encountered in dairy matrices, including AMP, AMX, CEX, PCN, DOX, as well as glucose as a representative non-antibiotic interferent. All analytes were examined at identical concentrations (62.5–1000 nM) to ensure a rigorous and unbiased comparison of interfacial responses.

**Fig. 3:**
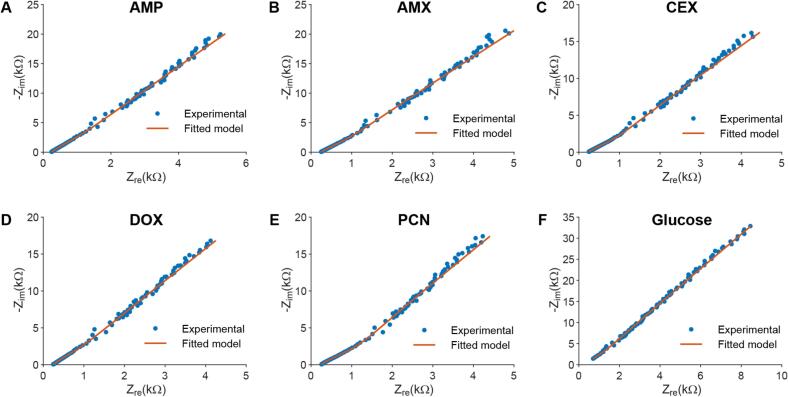
The Nyquist plots of the proposed sensors for different 62.5 nM antibiotic samples. The blue dots represent the experimental data, while the red lines correspond to the fitted data using the Randles equivalent circuit model shown in Fig. 1. (For interpretation of the references to colour in this figure legend, the reader is referred to the web version of this article.)

As illustrated by the representative Nyquist plots recorded at 62.5 nM (Fig. 3), tetracycline produces a markedly larger semicircle diameter than any of the competing species, corresponding to a substantially higher charge-transfer resistance. Even at the lowest tested concentration, the R_ct_ value obtained for tetracycline (66,209 Ω) is approximately five times higher than that observed for CEX and PCN (∼13,500 Ω), more than twice that of glucose, DOX, and AMX (31,606, 27,618, and 25,563 Ω, respectively), and still significantly higher than that of AMP (44,035 Ω). These pronounced differences indicate that the Mn:ZnS-CH interface responds preferentially to tetracycline, even at low concentrations relevant to residue monitoring.

**Fig. 4:**
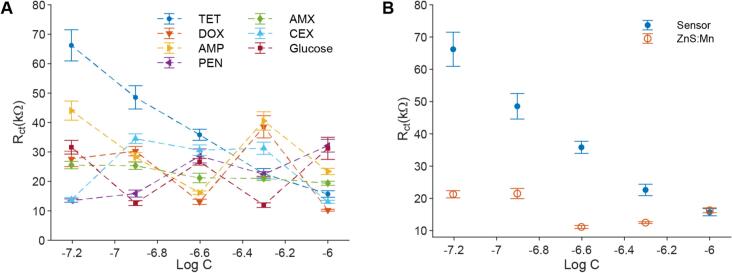
(A) Change in charge-transfer resistance R_ct_ as a function of the logarithm of concentration logC for the proposed biosensor exposed to different antibiotics and glucose. (B) Comparison of the R_ct_ responses toward tetracycline obtained using Mn:ZnS-CH-modified IDEs and Mn-doped ZnS-only IDEs, demonstrating the broader dynamic range and stronger concentration-dependent response provided by the chitosan-containing interface. Error bars represent the standard deviations of three independent measurements.

The discrimination trend is further quantified in Fig. 4A, where R_ct_ is plotted as a function of logC for each analyte. Tetracycline exhibits a steep, monotonic, and highly reproducible concentration-dependent decrease in R_ct_, accompanied by narrow error bars across the entire tested range. In contrast, the other antibiotics exhibit substantially flatter slopes, irregular trends, or non-monotonic responses, often accompanied by substantial scatter in the measured R_ct_ values. These behaviors suggest weak or unstable interactions with the sensing interface and support the use of slope, linearity, and response magnitude as criteria for discriminating tetracycline from potential interferents.

The regression analysis summarized in Table 3 provides quantitative support for these observations. Tetracycline shows both a large slope magnitude (−4.22 × 10^4^ Ω per logC) and excellent linearity (R^2^ = 0.98), confirming a predictable and concentration-dependent modulation of the interfacial charge-transfer pathway. In comparison, AMX, AMP, DOX, and CEX exhibit much smaller slopes and poor correlation coefficients (R^2^ = 0.12–0.89), indicating limited analytical sensitivity and weak coupling to the sensing surface. CEX displays virtually no linear relationship (R^2^ = 0.005), while glucose shows negligible correlation (R^2^ = 0.0005), confirming minimal electrochemical interaction with the Mn:ZnS-CH layer. PCN exhibits moderate linearity (R^2^ = 0.75), but its positive slope and lower absolute R_ct_ values clearly distinguish its response mechanism from that of tetracycline. Taken together, these results indicate that the Mn:ZnS-CH-modified IDEs provide good analytical discrimination against the tested interferents, although the platform should be described as exhibiting a preferential tetracycline response rather than absolute molecular selectivity.

**Table 3:** The fitting functions of sensors with different analytes.

	Linear fitting function of R_ct_ (Ω)	R^2^	Norm of residuals
Proposed sensors- TET	−4.22 × 10^4^logC-2.41 × 10^5^	0.98	5820
Proposed sensors- DOX	−0.88 × 10^4^logC-0.34 × 10^5^	0.12	22,430
Proposed sensors- AMP	−0.96 × 10^4^logC-0.33 × 10^5^	0.15	21,480
Proposed sensors- PCN	1.46 × 10^4^logC + 1.19 × 10^5^	0.75	7979
Proposed sensors- AMX	−0.55 × 10^4^logC-0.14 × 10^5^	0.89	1824
Proposed sensors- CEX	−0.15 × 10^4^logC + 0.15 × 10^5^	0.005	20,670
Proposed sensors- Glucose	−0.48 × 10^4^logC + 0.2 × 10^5^	0.0005	19,630
(Mn:ZnS) based sensors - TET	−0.63 × 10^4^logC-0.25 × 10^5^	0.39	7524

The origin of this pronounced discrimination can be attributed to the complementary interaction between tetracycline and the Mn:ZnS-CH hybrid interface. Tetracycline possesses β-diketone, enolic, and amide functional groups, which can participate in multiple binding interactions with the sensing surface. In parallel, the chitosan matrix provides an amine- and hydroxyl-rich environment that promotes hydrogen bonding and secondary coordination with tetracycline's aromatic and carbonyl functionalities, further stabilizing its adsorption at the electrode surface (Nguyen & Tran, 2024).

**Fig. 5:**
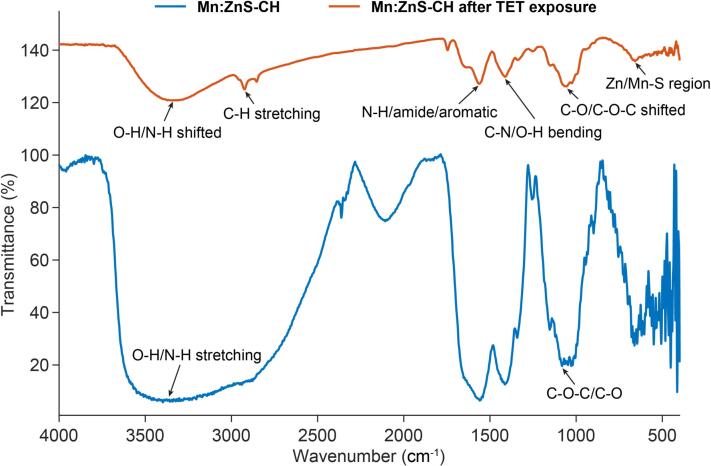
FTIR spectra of the Mn:ZnS-CH sensor before and after exposure to TET. The spectrum recorded after TET exposure was vertically offset for visual clarity.

As supported by the Fourier-transform infrared (FTIR) spectra in Fig. 5, the pristine Mn:ZnS-CH sensor exhibited characteristic chitosan-related bands, including the broad O-H/N-H stretching vibration at around 3350–3500 cm^−1^, amide-related vibrations in the 1420–1590 cm^−1^ region, and C-O-C vibrations near 1000–1100 cm^−1^, consistent with previous reports on chitosan-based materials (Bonfante-Alvarez et al., 2018; Nguyen et al., 2026). After exposure to TET, the broad O-H/N-H band shifted to a lower wavenumber, while the C=O and C-O-C related regions also showed noticeable changes. These spectral variations suggest the formation of hydrogen bonding between TET and the hydroxyl/amine groups of chitosan, which is consistent with reported interactions between tetracycline and polysaccharide- or biopolymer-based adsorbents (Zhu et al., 2020).

In addition to hydrogen bonding, TET can coordinate with surface Zn^2+^/Mn-related sites through its oxygen-rich functional groups, particularly the β-diketone and phenolic/enolic moieties. Similar metal–tetracycline coordination and inner-sphere surface complexation have been reported previously, where tetracycline interacts with metal-containing surfaces through carbonyl, phenolic, and diketone groups (Zhao et al., 2014). In contrast, β-lactam antibiotics lack comparable multidentate chelating motifs and therefore interact much more weakly with the Mn:ZnS-CH interface. Taken together, the FTIR changes shown in Fig. 5 and the selective electrochemical response indicate that TET adsorption on the Mn:ZnS-CH surface is governed by the combined effects of hydrogen bonding, electrostatic interaction, and metal-ligand coordination (Zhao et al., 2014; Zhu et al., 2020). These interfacial interactions enhance local dielectric polarization and raise the interfacial charge-transfer barrier, resulting in the distinctive impedance response observed for tetracycline.

To further elucidate the role of chitosan in achieving preferential tetracycline response and enhanced sensitivity, the performance of the Mn:ZnS-CH sensor was directly compared with that of a sensor fabricated using Mn-doped ZnS nanoparticles alone (Fig. 4B). At 62.5 nM tetracycline, the Mn:ZnS-CH-modified IDE exhibits an R_ct_ value of 66,209 Ω, more than three times higher than that obtained with the Mn:ZnS-only electrode (21,275 Ω). Even at higher concentrations (1000 nM), where impedimetric responses often converge, the Mn:ZnS-CH system maintains a substantially broader dynamic range (15,704–66,209 Ω) compared with the narrow response window of the Mn:ZnS-only system (16,297–21,275 Ω). This expanded dynamic range directly contributes to the superior linearity and analytical reliability of the Mn:ZnS-CH platform (R^2^ = 0.96), whereas the Mn:ZnS-only sensor shows limited predictive power (R^2^ ≈ 0.38). The sensitivity enhancement is further reflected in the calibration slopes, with the Mn:ZnS-CH sensor exhibiting a slope of −4.39 × 10^4^ Ω per logC, approximately seven times greater than that of Mn: ZnS alone (−6.3 × 10^3^ Ω per logC). This marked improvement highlights the critical role of chitosan as an active interfacial component rather than a passive binder, as it promotes nanoparticle dispersion, increases analyte accessibility, and enables multiple interaction pathways necessary for effective charge-transfer modulation.

The contribution of chitosan may also be influenced by its pH-sensitive behavior. Chitosan-containing networks are known to exhibit pH-dependent gelation, hydration, porosity, and molecular loading/release characteristics, which can affect the accessibility of functional groups and analyte-surface interactions. In the Mn:ZnS-CH interface, protonation/deprotonation of amino groups may modulate surface charge, hydrogen bonding, chelation, and interfacial hydration, thereby influencing tetracycline adsorption and the resulting impedance response (Alimirzaei et al., 2017; Ayazi et al., 2020). In this study, the Mn:ZnS-CH layer was prepared under fixed fabrication conditions, and all sensing and interference measurements were conducted in the same diluted-milk environment to ensure consistent comparison. Therefore, the observed analytical discrimination toward tetracycline reflects the optimized interface's response under controlled conditions of pH 6.08. Further optimization of the fabrication pH and the sensing medium pH may provide an additional route to tune the response and improve the discrimination capability of chitosan-based impedimetric sensors, and will be the subject of future work.

Taken together, the clear separation in R_ct_ values, the distinct calibration slopes, and the pronounced divergence in correlation coefficients between tetracycline and the interfering species collectively confirm the intrinsic selectivity of the Mn:ZnS-CH biosensor toward tetracycline. The ability to maintain this high degree of molecular discrimination in diluted milk further demonstrates the robustness of the sensing interface against matrix-induced interference. These results establish the Mn:ZnS-CH-modified IDE platform as a highly selective and analytically reliable impedimetric system for tetracycline screening in complex dairy matrices.

### 3.3 Analytical validation, reproducibility, and stability in tetracycline-spiked Milk samples

The pronounced discrimination and concentration-dependent impedance response observed in Section 3.2 indicate that the Mn:ZnS-CH-modified IDE platform can reliably discriminate tetracycline from other antibiotics in a complex dairy environment. However, for practical food analysis, molecular selectivity alone is insufficient; the sensing response must also remain accurate, reproducible, and stable when applied to real samples. To address these requirements, the analytical performance of the proposed platform was systematically evaluated using tetracycline-spiked milk samples, with particular emphasis on accuracy, reproducibility across independently fabricated electrodes, and temporal stability.

Analytical validation was performed by spiking diluted milk samples with known concentrations of tetracycline, followed by converting the experimentally measured charge-transfer resistance values to analyte concentrations using the calibration model established in Section 3.1. The validation results are summarized in Table 4. For milk samples spiked at 1.0 × 10^−7^ M TET, the calculated logarithmic concentration (logC) was −7.03, corresponding to a relative error of 3.11%. Similarly, for samples containing 3.5 × 10^−7^ M TET, a logC value of −6.44 was obtained, yielding an error of 2.08%. Because tetracycline was spiked after milk dilution, these results primarily validate the accuracy of tetracycline quantification in the 1:30 diluted-milk matrix rather than the extraction recovery of tetracycline pre-bound to native milk proteins or fat components. The close agreement between spiked and calculated concentrations confirms that the calibration model remains reliable in the chemically complex milk matrix. Importantly, the low analytical errors observed (<5%) indicate that the Mn:ZnS-CH sensing interface effectively mitigates matrix-induced interferences arising from proteins, lipids, lactose, and endogenous ionic species commonly present in dairy products. Although the present study was conducted under controlled laboratory conditions rather than as an on-site field trial, commercial milk was used as a real food matrix to evaluate the practical applicability of the proposed sensor. The dilution strategy and matrix-matched calibration helped minimize the influence of milk components on the impedance response, supporting reliable quantification in a complex dairy matrix. Nevertheless, further validation using naturally contaminated milk samples and on-site measurements at dairy farms or processing facilities will be required for future field deployment. The ability to achieve accurate quantification without extensive sample pretreatment highlights the robustness of the IDE-based impedimetric approach for real-sample analysis.

**Table 4:** The validation data of spiked samples.

C (M)	logC	Fitted R_ct_ (Ω)	Estimated logC	Error (%)
1.00 × 10^−7^	−7	55,874	−7.03	3.11
3.50 × 10^−7^	−6.46	30,745	−6.44	2.08

Beyond analytical accuracy, the reproducibility of the sensing platform was evaluated by independently fabricating Mn:ZnS-CH-modified electrodes on different days under identical conditions using Mn:ZnS-CH materials obtained from different synthesis batches. The relative standard deviations (RSDs) derived from TET-induced R_ct_ responses consistently ranged from 5 to 10%, demonstrating good batch-to-batch and electrode-to-electrode reproducibility and uniform interfacial properties of the sensing layer. In addition, consecutive impedance measurements on the same electrode showed negligible signal fluctuations, confirming good short-term repeatability and stable electron-transfer behavior during continuous operation. These results suggest that variations arising from the Mn:ZnS-CH synthesis and electrode-coating processes did not significantly affect the analytical response of the proposed sensor under the tested conditions.

Furthermore, the stability of the Mn:ZnS-CH sensing interface was evaluated from two perspectives: the storage stability of the nanocomposite material prior to sensor fabrication and the response stability of the fabricated sensors over time. To assess whether prolonged storage affected the electrochemical properties of the material, the same Mn:ZnS-CH nanocomposite batch used for the initial sensor characterization was stored under ambient conditions. After two months of storage, this batch was used to fabricate a new set of sensors following the same coating protocol. The initial Rct value obtained from these sensors in the present stability study was 33,330 ± 4765.17 Ω, which was in close agreement with the baseline Rct value reported in Table 1 for sensors fabricated from the same batch at the beginning of the study (33,655 ± 1878.81 Ω). This close agreement indicates that two months of material storage did not significantly affect the electrochemical properties of the Mn:ZnS-CH nanocomposite.

The response stability of the fabricated sensors was then examined by exposing independently prepared sensors to a fixed TET concentration of 250 nM. The Rct response was monitored at different time points after fabrication, including 0 min, 30 min, 120 min, 1 day, 3 days, 1 week, 2 weeks, 3 weeks, and 4 weeks. At each time point, three consecutive electrochemical impedance spectroscopy (EIS) measurements were recorded. The average R_ct_ values, standard deviations, and relative changes from the initial response are summarized in Table 5.

**Table 5:** Fitted charge-transfer resistance R_ct_ of sensors exposed to 250 nM TET at different time points after fabrication.

Time	R_ct_-AVE (Ω)	SD (Ω)	ΔR_ct_ (%)
0 min	33,330	4765.17	
30 min	30,687.56	3142.79	7.93
120 min	33,243	1009.36	0.26
1 day	33,634.78	2260.33	0.91
3 days	32,890	1791.00	1.32
1 week	32,701.75	2972.06	1.88
2 weeks	32,632	3205.35	2.09
3 weeks	34,695	913.91	4.10
4 weeks	31,359.67	1226.51	5.91

In Table 5, the relative signal variation was quantified using the equation:

(3) $$∆R_{\mathrm{ct}}=\frac{R_{\mathrm{ct}}-R_{\mathrm{ct},0}}{R_{\mathrm{ct},0}}\times 100$$

where R_ct,0_ corresponds to the initial value at *t* = 0. As shown in Table 5, the sensor exhibited only minor variations in impedance response during storage. The relative change in Rct remained below 8% throughout the tested period, with the highest variation observed after 30 min. After longer storage times, the signal became more stable, with ΔRct values of 0.91% and 1.32% after 1 and 3 days, respectively. Importantly, the data further demonstrate that the sensor retained a stable response over extended storage, with ΔRct values of only 1.88%, 2.09%, 4.10%, and 5.91% after 1, 2, 3, and 4 weeks, respectively. These results demonstrate that the Mn:ZnS-CH sensing interface maintained a reproducible impedance response over time, with no pronounced signal drift during the four-week monitoring period. Overall, these results demonstrate that the Mn:ZnS-CH sensor exhibited good storage stability and response reproducibility, supporting its potential use for practical TET detection.

Taken together, the validation results support the applicability of the Mn:ZnS-chitosan impedimetric biosensor for tetracycline detection in dairy matrices. Rather than serving as a confirmatory regulatory method, the proposed IDE-EIS platform is better positioned as a rapid, low-cost, and simple preliminary screening tool for identifying samples that may require further chromatographic confirmation. When combined with the high sensitivity and analytical discrimination reported in earlier sections, these results highlight the practical potential of the proposed platform for rapid, on-site screening of antibiotic residues in food-safety and quality-assurance applications. Nevertheless, because the present validation was performed using spiked commercial milk under controlled laboratory conditions, further studies using naturally contaminated samples, larger sample sets, and on-site measurements are needed to confirm field applicability.

Finally, a comparison of representative tetracycline-sensing platforms reported for milk analysis is summarized in Table 6. These reported platforms include aptamer-based EIS and voltammetric biosensors, photoelectrochemical aptasensors, lateral-flow strip sensors, graphene field-effect transistor sensors, direct electrochemical detection using gold nanostructured electrodes, and paper-based colorimetric devices. Most previously reported systems rely on aptamer-based molecular recognition combined with complex surface functionalization or signal-labeling strategies. Other approaches improve portability or simplicity through lateral-flow or paper-based formats, but may sacrifice quantitative precision or detection sensitivity, whereas direct electrochemical methods can require additional complexation steps or sample pretreatment. While such approaches can achieve ultralow detection limits, they often suffer from limited stability, higher fabrication complexity, and increased cost. In contrast, the present Mn:ZnS-chitosan biosensor employs a label-free, non-biological recognition strategy based on defect-engineered nanomaterials embedded in a biopolymer matrix, enabling robust and reproducible charge-transfer modulation in protein- and fat-rich media. Although the proposed sensor does not provide the lowest LOD among all reported systems, it offers a practical balance of sensitivity, simplicity, aptamer-free fabrication, and reliable performance in a diluted milk matrix. This material design is consistent with the broader trend of functional nanomaterial-enabled biosensing, where tailored electrical conductivity, surface reactivity, and interfacial properties are used to enhance signal generation and sensing performance. Recent studies on graphene-based micro/nano-systems further illustrate how engineered nanomaterials can support advanced biosensing functions through their high conductivity, large surface area, and strong coupling with functional materials (Ebrahimi et al., 2024). In the present platform, Mn doping is expected to introduce defect-related electronic states that facilitate charge transport, while chitosan improves nanoparticle dispersion, film stability, and tetracycline-surface interactions. This combination of simplicity, stability, and analytical performance positions the proposed platform as a practical and scalable alternative for routine tetracycline monitoring in dairy products.

**Table 6:** Comparison of representative biosensors for tetracycline detection in milk.

Study	Recognition Element	Transduction Method	Linear Range	LOD	Milk Sample Performance	Key Limitations / Remarks
(Lu et al., 2021)	None / colorimetric reaction	Paper-based colorimetric device	2.25–225 μM	2.25 μM	Tested in 18 milk samples; recovery 88–113%	Low-cost, portable, and equipment-free, but lower sensitivity and limited quantitative precision compared with electrochemical methods
(Raykova et al., 2023)	Fe(III)-assisted tetracycline complexation	DPV on a gold nanostructured electrode	Responsive up to 2 mM	345 nM in buffer; 931 nM in milk	Detection demonstrated in spiked whole milk after protein removal	Simple direct electrochemical strategy, but requires acidic Fe(III) complexation and sample pretreatment; relatively high LOD in milk
(Dou et al., 2024)	Aptamer	Graphene field-effect transistor	Not specified	2.073 pM and 100 pM, depending on detection mode	Applied to skim milk with good recovery	Highly sensitive and miniaturized, but requires aptamer-modified graphene FET fabrication and specialized electronic readout
(Le et al., 2016)	Aptamer	EIS (Impedimetric)	10–3000 ng mL^−1^ (∼22–6700 nM)	10 ng mL^−1^ (∼22 nM)	Recovery: 88.1–94.2% Sensitivity: 98% Specificity: 100%	Requires aptamer immobilization; moderate sensitivity; relatively long assay time (15 min)
(Mohammad-Razdari et al., 2020)	Aptamer	EIS on PGE/rGO	10^−16^–10^−6^ M	3 × 10^−17^ M	Recovery: 92.8–102.1%	Ultrahigh sensitivity but involves complex surface optimization and biological receptor instability
(Malecka-Baturo et al., 2022)	Ferrocene-labeled aptamer	Square-wave voltammetry	Not specified	0.16 nM (buffer); 0.20 nM (milk)	Successful detection in spiked milk	Requires redox labeling, Au electrodes, and covalent immobilization
(Zhai et al., 2025)	Broad-spectrum aptamer	Lateral flow strip (LFS)	1–300 nM	0.33 nM	Recovery: 93.6–106.2%	Rapid and portable, but semi-quantitative and limited signal precision
(Xu, 2024)	Aptamer	Photoelectrochemical (PEC)	5–300 nM	1.24 nM	Acceptable accuracy in milk	Requires photoactive heterojunctions and light source
This work	None (non-biological)	Label-free EIS (Impedimetric)	62.5–1000 nM	2.47 nM	<5% error; good analytical discrimination and stability.	Simple fabrication, no aptamers or labels; robust in complex milk matrix

## 4 Conclusion

In this study, the feasibility of a Mn:ZnS-chitosan-modified IDE-EIS platform for rapid screening of tetracycline in milk was demonstrated. The sensor relies on charge-transfer modulation at a defect-engineered nanomaterial/biopolymer interface, avoiding the need for aptamers, enzymes, or labeling steps. The IDE-EIS configuration produced a clear signal-on response, enabling reliable quantification over a linear concentration range of 62.5–1000 nM, with a limit of detection of 2.47 nM and high analytical accuracy in tetracycline-spiked milk samples (errors below 5%). These results confirm the proposed approach's ability to operate effectively in complex dairy matrices without extensive sample pretreatment. The sensing platform exhibited good analytical discrimination against the tested interferents, along with acceptable reproducibility and short-term stability across independently fabricated electrodes, supporting its robustness for routine analytical use. Importantly, while chromatographic techniques such as HPLC remain the reference methods for regulatory analysis of antibiotic residues, the present IDE-based impedimetric approach is intended as a complementary, rapid, and cost-effective screening tool for preliminary assessment of tetracycline contamination in milk prior to confirmatory analysis when required. Future work should include naturally contaminated samples, larger sample sets, and on-site testing to further validate field applicability. Overall, the results highlight the potential of IDE-EIS methodologies integrated with functional nanomaterial interfaces for practical food-safety monitoring and broader contaminant screening in complex food matrices.

## CRediT authorship contribution statement

**Son Hai Nguyen:** Writing – review & editing, Supervision, Project administration, Methodology, Investigation, Funding acquisition, Formal analysis, Conceptualization. **Huy Quang Nguyen:** Formal analysis, Data curation. **Mai Thi Tran:** Writing – original draft, Visualization, Validation, Investigation, Formal analysis, Conceptualization.

## Declaration of competing interest

The authors declare that they have no known competing financial interests or personal relationships that could have appeared to influence the work reported in this paper.

## Data Availability

Data will be made available on request.
